# Apolipoprotein E gene polymorphism and dyslipidaemia in adult Asian Indians: A population based study from Calcutta, India

**DOI:** 10.4103/0971-6866.45000

**Published:** 2008

**Authors:** Mithun Das, Susil Pal, Arnab Ghosh

**Affiliations:** 1 Post Graduate Department of Anthropology, Sree Chaitanya College, Habra, West Bengal, India; 2 Human Genetic Engineering Research Centre, Calcutta, India; 3 Biomedical Research Laboratory, Department of Anthropology, Visva Bharati University, Santiniketan, West Bengal, India

**Keywords:** Apo E polymorphism, Asian Indians, dyslipidaemia, metabolic, syndrome, obesity

## Abstract

**AIM:**

The study was aimed to determine the association of Apolipoprotein E (apo E) gene polymorphisms on lipid levels in Asian Indian population.

**METHODS:**

A total of 350 (184 males and 166 females) adult (30 years and above) Asian Indians of Calcutta and suburb participated in the study. Anthropometric measures, lipids profiles, and blood glucose measures were collected. Out of 350 subjects, a sample of 70 individuals was selected randomly for genotyping after adjusting for age and sex. The apo E gene polymorphisms were determined by agarose gel electrophoresis.

**RESULTS:**

The apo E polymorphism showed significant association with dyslipidaemia (*P*=0.0135) with ε3/4 combination has had the highest occurrence of dyslipidaemia and metabolic syndrome (MS) followed by ε4/4 <ε3/3 <ε2/4 <ε2/3 in decreasing order.

**CONCLUSIONS:**

The ε4 allele of apo E gene independent of other risk factors is associated with dyslipidaemia in particular with low HDLc and high TC: HDLc ratio.

## Introduction

Apolipoprotein E (apoE) plays a key role in the metabolism of cholesterol and triglyceride by serving as a receptor binding ligand mediating the clearance of chylomicron and remnants of very low-density lipoprotein cholesterol from plasma.[1–4] Three different apoE alleles (ε2, ε3, and ε4) on chromosome number 19 are responsible to encoding three different apo E isoform (apo E2, apo E3, and apo E4 respectively), resulting in six different genotypes (ε2/2, ε3/3, ε4/4, ε2/3, ε2/4, and ε3/4).[5,6] It has been estimated that apo E polymorphisms may account for 2-16% of the variability of LDL cholesterol levels, a contribution incomparable with any other gene in the general population.[2,3] Compared with ε3 homozygotes, carriers of the ε2 allele, which has defective receptor-binding ability, have lower circulating cholesterol levels and higher triglyceride levels, whereas carriers of the ε4 allele appear to have higher plasma levels of total and low-density lipoprotein cholesterol.[1] Since the first demonstration of apoE gene polymorphism on dysbetalipoproteinemia,[7] numerous studies have investigated the relation between apoE genotypes and coronary heart disease (CHD).[7–9] Recent evidence also indicates that apoE may play additional role in the development of CHD.[2,10–13]

Globally, the apoE locus shows substantial allelic variation with ranges 0-20% for ε2, 60-90% for ε3 and 10-20% for ε4 alleles[14–18] with some exception. In Asian Indians, studies have investigated the allele frequencies with ranges 0.031-0.094 for ε2; 0.803-0.968 for ε3 and 0.000-0.133 for ε4.[19] Frequency of apoE ε3 allele was found to be high (0.913) in people of north India.[17] In another study on Asian Indians, individuals with at least a ε4 allele were considered at risk to develop premature Myocardial Infarction (MI), independent of other risk conventional risk factors.[20] Most of these studies are either from the north, west, central, and southern part of India.[15,7–32] Studies from eastern part of India are lacking. The present study is an attempt to investigate the apo E gene polymorphism and its association with dyslipidemmia in people belongs to the eastern part of India.

## Materials and Methods

### Study population

The data were collected from adult men and women aged 30 years and above from Calcutta and suburb. A total of 350 individuals (184 males and 166 females) participated in the study. Prior to participation, public advertisement was given about the study with the help of the council officials. An arbitrary list was made to conduct the study. Individuals were selected randomly after their response through local advertisement. The response rate was quite high (~85%). The institute ethical committee has had approved the study. Written consent was obtained from participants prior to actual commencement of the study.

### Anthropometric measurements

Anthropometric variables namely height, weight, circumference of waist (WC) and hip were taken using standard techniques.[33] Height was measured to nearest 0.1 cm with a standard anthropometer and weight to the nearest 0.5 kg with a portable weighing machine in light clothing. Waist and hip circumferences were measured to the nearest 0.1 cm using non-elastic tape. The body mass index (BMI in kg/m^2^) and waist-hip ratio (WHR) was calculated accordingly.

### Biochemical estimations

A 7 ml of venous blood was drawn from each individual after an overnight fast of ≥12 h for estimation of metabolic variables. Lipids profile namely the total cholesterol (TC), triglycerides (TG), high density lipoprotein cholesterol (HDLc), low density lipoprotein cholesterol (LDLc), and very low density lipoprotein cholesterol (VLDLc) were taken into consideration. Values of LDLc were estimated using standard formula (LDLc= TC - (HDLc + TG/5)) and VLDLc as TG/5 provided TG was < 400 mg/dl. Otherwise, TG was estimated directly. Fasting (FBG), post prandial blood glucose (PPBG) (2 h after meal) and TC: HDLc ratio was also considered. All biochemical analyses were done at the ’Human Genetic Engineering Research Center (HGERC), Calcutta and were measured in mg/dl (mg %) unit. Subjects with one or more of the followings were considered as dyslipidaemic (post entry): TC ≥ 240 mg/dl;[34] TG ≥ 200 mg/dl;[35] and TC: HDLc ratio ≥ 4.4.[36]

### Genotyping

DNA was extracted from blood using QI Aamp Kit (QIAGEN, Germany). Apo E isoforms were amplified by polymerase chain reaction with a thermal cycler (Bio Rad, USA) and specific oligonucleotides as described.[37] The amplified fragments were digested with the enzyme *HhaI* overnight at 37°C and separated on a 4% agarose (Promega, USA) gel electrophoresis with ethidium bromide staining and visualized under UV spectrophotometer.

### Statistical analyses

Descriptive statistics such mean and standard deviation (SD) of anthropometric and biochemical measures was calculated by sex and the sex differences for these variables were calculated using unpaired *t* test. Differences in genotype frequency were examined by χ^2^ analyses. Analysis of variance (ANOVA) was undertaken to see the differences of obesity and metabolic variables across the apo E genotypes.

All statistical analyses were computed using the SPSS (PC + version10.0).[38] A *P* value of < 0.05 (two tailed) was considered as significant.

## Results

The mean and SD (unadjusted for age) of anthropometric, lipids profile and blood glucose variables by sexes are presented in Table 1. There existed no significant sex differences for variables except for age, TC and FBG. Since, age differed significantly, age and sex matched 70 subjects were selected randomly to study the association of apo E polymorphism and dyslipidaemia.

**Table 1:** Characteristics of the study population (*n*=350)

	Male *n*=184)		Female (*n*=166)		
			
Variables	Mean	SD	Mean	SD	*P value*
Age (yrs)	54.04	12.40	48.48	11.57	<0.001
BMI (kg/m^2^)	22.37	4.09	23.20	4.37	0.066
WC (cm)	89.81	10.04	88.90	9.69	0.392
TC (mg/dl)	205.33	27.90	199.46	22.84	0.033
TG (mg/dl)	143.74	27.48	139.98	22.56	0.165
HDLc (mg/dl)	44.34	4.80	44.36	4.65	0.970
LDLc(mg/dl)	132.23	27.42	127.13	23.03	0.062
VLDLc (mg/dl)	28.77	5.50	28.03	4.51	0.175
TC: HDLc	4.73	1.10	4.57	0.88	0.134
FBG (mg/dl)	93.15	23.50	88.57	16.89	0.039
PPBG^#^ (mg/dl)	130.64	56.13	120.11	38.25	0.089

BMI= body mass index, WC= circumference of waist, TC= total cholesterol, TG= triglyceride, HDLc= high density lipoprotein cholesterol, LDLc= low density lipoprotein cholesterol, VLDLc= very low density lipoprotein cholesterol, FBG= fasting blood glucose, PPBG= post prandial blood glucose

# Male (*n*=139) and Female (*n*=114)

The distribution of risk variables by apo E genotypes are presented in Table 2. It was observed that ε3/4 individuals have relatively higher mean value for BMI, WC, TC, LDLc, low HDLc, and TC: HDLc ratio as compared to rest of the genotypes. Homozygous for ε4 allele on contrary have had relatively higher TG value whereas, individual homozygous for ε3 allele have had relatively higher FBG and PPBG compared to other groups. One way ANOVA revealed significant difference across the groups for HDLc (*P*=0.039) and TC: HDLc ratios (*P*=0.013) only. Individuals heterozygous for ε3/4 alleles have had lowest average HDLc and highest TC: HDLc ratio followed by ε4/4 individuals and ε3/3 individuals. The allele frequencies are as follows: ε2 = 0.100; ε3 = 0.771, and ε4 = 0.129 and the genotype frequencies are: ε2/3 = 0.171; ε2/4 = 0.029; ε3/4 = 0.114; ε3/3 = 0.629, and ε4/4 = 0.057 (not shown in Tables).

**Table 2:** Distributi on of risk variables by apoE genotypes

	apo E genotypes (*n*=70)					
		
Variables	ε2/3	ε2/4	ε3/4	ε3/3	ε4/4	*P value for ANOVA*
	(*n*=12)	(*n*=2)	(*n*=8)	(*n*=44)	(*n*=4)	
BMI (kg/m^2^)	21.06	22.40	24.12	22.85	22.00	0.616
	(5.44)	(4.66)	(3.59)	(4.13)	(4.74)	
WC (cm)	85.08	85.50	94.37	88.66	91.75	0.417
	(15.43)	(7.78)	(10.01)	(9.40)	(13.33)	
TC (mg/dl)	196.17	163.50	227.75	209.48	216.00	0.084
	(18.93)	(7.78)	(27.39)	(36.96)	(15.21)	
TG (mg/dl)	126.08	105.00	164.50	144.86	169.75	0.064
	(32.53)	(15.56)	(42.33)	(37.53)	(39.19)	
HDLc (mg/dl)	46.25	49.00	38.13	43.52	39.50	0.039
	(6.45)	(4.24)	(8.66)	(6.24)	(2.38)	
LDLc (mg/dl)	124.67	93.50	156.88	137.05	142.50	0.070
	(19.61)	(9.19)	(26.13)	(35.22)	(14.25)	
TC: HDLc	4.36	3.35	6.26	4.97	5.49	0.013
	(0.99)	(0.44)	(1.60)	(1.39)	(0.64)	
FBG (mg/dl)	86.25	78.00	93.25	95.98	93.00	0.733
	(11.57)	(2.83)	(19.46)	(29.74)	(27.82)	
PPBG^#^ (mg/dl)	107.17	102.00	115.00	140.16	131.67	0.702
	(5.71)	-	(23.61)	(69.39)	(51.87)	
	(*n*=6)	(*n*=1)	(*n*=5)	(*n*=32)	(*n*=3)	

**Figure 1:**
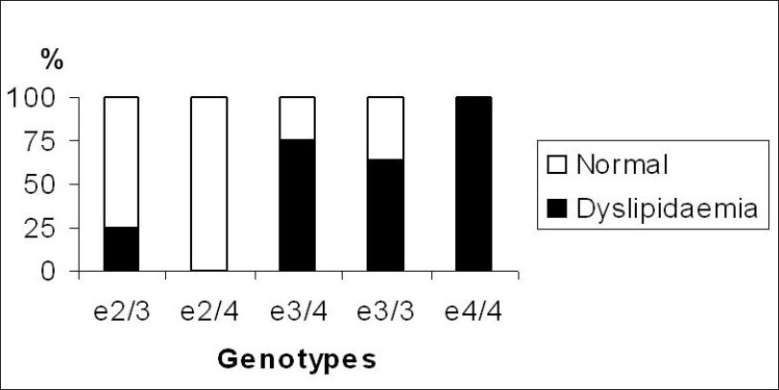
Incidence of dyslipidaemia by apo E genotypes

The incidence of dyslipidaemia by apo E genotypes is presented in Table 3. The Chi-square revealed significant (*P*=0.0135) group differences in incidence of dyslipidaemia by genotypes. Incidence percentage of dyslipidaemia is shown in Figure 1. The incidence is 25% for ε2/3, 75% for ε3/4, 63.64% for ε3/3 and 100% for ε4/4 genotypes.

**Table 3:** Occurrence of dyslipidaemia according to apo E genotypes

	apo E genotypes					
		
	ε2/3	ε2/4	ε3/4	ε3/3	ε4/4	Total
Dyslipidaemia	3	0	6	28	4	41
Normal	9	2	2	16	0	29
Total	12	2	8	44	4	70

χ^2^ _(df = 4)_ = 12.58, *P* = 0.0135

## Discussion

The present investigation was aimed to examine whether significant association does exist between apo E gene polymorphism and dyslipidaemia in adult Asian Indians living in the eastern part of India. Previous studies from other parts of India have had reported the allele frequencies ranges from 0.031 to 0.094 for ε2; 0.803-0.968 for ε3, and 0.000-0.133 for ε4. The present study from Eastern India have had shown an almost similar frequency of ε4 allele. It has been found that individuals with ε3/4 combination as well as homozygous for ε4 allele (ε4/4) have significantly higher occurrence of dyslipidaemia compare to other genotypes combinations. The occurrence of dyslipidaemia as found in the study could be summarized in the following way (decreasing order): ε3/4 < ε4/4 < ε3/3< ε2/4< ε2/3. In a similar study on Omani dyslipidaemic patients, it was observed that thirty-one percent of patients with CHD had the APOE*4 allele compared to 26% with the APOE*3 allele, but this difference was not significant.[39] In another study, it was observed that the prevalence of the ε4-containing phenotypes were significantly higher in ischemic patients and that apo ε4 is an independent risk factor associated with an altered lipid profiles.[40] In the Ouro Preto admixed population in Brazil, it demonstrated that the presence of APOE*2 can confer a protective effect, whereas the presence of APOE*4 implies an enhanced risk for dyslipidemia.[41]

The major limitation of the study was that it was performed on a small sample size and therefore is not representative of Asian Indian population. Owing to ethnic and cultural heterogeneity in Asian Indian population, it is imperative to study the ethnic groups collectively, possibly through collaborative research. This will give more insight into proper understanding of the association lying between apo E gene polymorphisms and dyslipidaemia among Asian Indians.

## Conclusion

It seems that the allele frequency of ε4 is quite high in the present studied population as compare to most of the other parts of India. Prevalence of dyslipidaemia is significantly high among individuals with ε3/4 and ε4/4 genotypes compare to other combinations. It seems reasonable to argue that ε4 allele has an independent effect on lipids metabolism particularly increased TC: HDLc ratio causing dyslipidaemia in the study population.
